# Construction of a high-density genetic map using specific length amplified fragment markers and identification of a quantitative trait locus for anthracnose resistance in walnut (*Juglans regia* L.)

**DOI:** 10.1186/s12864-015-1822-8

**Published:** 2015-08-18

**Authors:** Yufeng Zhu, Yanfei Yin, Keqiang Yang, Jihong Li, Yalin Sang, Long Huang, Shu Fan

**Affiliations:** College of Forestry, Shandong Agricultural University, No.61 Daizong Load, Taian, Shandong Provence 271018 P. R. China; Biomarker Technologies Corporation, Beijing, P. R. China

**Keywords:** *Juglans regia* L, Genetic map, Specific length amplified fragment sequencing, SLAF-seq, Anthracnose, *Colletotrichum gloeosporioides*, Quantitative trait locus, QTL

## Abstract

**Background:**

Walnut (*Juglans regia*, 2n = 32, approximately 606 Mb per 1C genome) is an economically important tree crop. Resistance to anthracnose, caused by *Colletotrichum gloeosporioides*, is a major objective of walnut genetic improvement in China. The recently developed specific length amplified fragment sequencing (SLAF-seq) is an efficient strategy that can obtain large numbers of markers with sufficient sequence information to construct high-density genetic maps and permits detection of quantitative trait loci (QTLs) for molecular breeding.

**Results:**

SLAF-seq generated 161.64 M paired-end reads. 153,820 SLAF markers were obtained, of which 49,174 were polymorphic. 13,635 polymorphic markers were sorted into five segregation types and 2,577 markers of them were used to construct genetic linkage maps: 2,395 of these fell into 16 linkage groups (LGs) for the female map, 448 markers for the male map, and 2,577 markers for the integrated map. Taking into account the size of all LGs, the marker coverage was 2,664.36 cM for the female map, 1,305.58 cM for the male map, and 2,457.82 cM for the integrated map. The average intervals between two adjacent mapped markers were 1.11 cM, 2.91 cM and 0.95 cM for three maps, respectively. ‘SNP_only’ markers accounted for 89.25 % of the markers on the integrated map. Mapping markers contained 5,043 single nucleotide polymorphisms (SNPs) loci, which corresponded to two SNP loci per SLAF marker. According to the integrated map, we used interval mapping (Logarithm of odds, LOD > 3.0) to detect our quantitative trait. One QTL was detected for anthracnose resistance. The interval of this QTL ranged from 165.51 cM to 176.33 cM on LG14, and ten markers in this interval that were above the threshold value were considered to be linked markers to the anthracnose resistance trait. The phenotypic variance explained by each marker ranged from 16.2 to 19.9 %, and their LOD scores varied from 3.22 to 4.04.

**Conclusions:**

High-density genetic maps for walnut containing 16 LGs were constructed using the SLAF-seq method with an F1 population. One QTL for walnut anthracnose resistance was identified based on the map. The results will aid molecular marker-assisted breeding and walnut resistance genes identification.

**Electronic supplementary material:**

The online version of this article (doi:10.1186/s12864-015-1822-8) contains supplementary material, which is available to authorized users.

## Background

Walnut (*Juglans regia* L., 2n = 32, approximately 606 Mb per 1C genome [1, 2]) is an economically important tree crop whose kernels very high nutritional values and is widely grown in many countries [3, 4]. Walnut anthracnose, caused by *Colletotrichum gloeosporioides*, is one of the most serious walnut diseases, which leads to sharp reductions in production [5]. To date, chemical control has been the main measure for disease control; however, its use is restricted because of environmental problems and drug resistance in the pathogens [6–8]. Therefore, resistance breeding is required. Genetic markers linked to resistance genes can improve the efficiency of breeding [9]. With the development of genetic markers, resistance gene analogs (RGAs) have been isolated from walnut, which indicated that anthracnose resistance in walnut was controlled by resistance genes (R genes) [10, 11]. A high-density genetic linkage map represents an important tool for quantitative trait locus (QTL) mapping, and QTLs for resistance to disease have been studied in many plants [12, 13]. Several genetic linkage maps have been constructed for walnut; however, QTLs associated with anthracnose resistance have not been reported.

In the past two decades, different molecular markers, including restriction fragment length polymorphisms (RFLP) [14], random amplified polymorphic DNA (RAPD) [15], simple sequence repeat (SSR) [16] and inter-simple sequence repeat (ISSR) [17], have been developed and used to evaluate genetic diversity of walnut. RFLP and RAPD have been used to construct walnut genetic maps. Fjellstrom et al. [1] first constructed a walnut genetic map using 42 RFLP markers covering 301.6 cM with an average marker spacing of 7.18 cM. Woeste et al. [18] obtained a walnut genetic map using 48 RFLP and 59 RAPD markers, based on a Backcross (BC) population with 49 individuals. The total marker number on these maps is generally limited and some of the mapped markers have no sequence information. Therefore, a high-density genetic linkage map for walnut is required that comprises a large number of markers with sufficient sequence information. In recent years, a bacterial artificial chromosome (BAC) library was used to develop single nucleotide polymorphism (SNP) markers in walnut [19, 2]. This approach is efficient to obtain more markers to increase the density of walnut maps.

The advent of next-generation sequencing (NGS), combined with the use of restriction enzymes, has proved valuable for the discovery, validation and assessment of a large number of genetic markers, which are essential to construct highly dense linkage maps, and to identify recombination breakpoints for QTL positions [20–22]. NGS led to the development of new methods, including reduced-representation sequencing using reduced-representation libraries (RRLs) or complexity reduction of polymorphic sequences (CRoPS) [23], genotyping by sequencing (GBS) [24] and restriction-site associated DNA sequencing (RAD-seq) [25]. RAD-seq is widely used for creating genetic linkage maps and SNP discovery in large plant species [26, 27]. Recently, the high-resolution method of specific length amplified fragment sequencing (SLAF-seq), which is based on RRLs and high-throughput sequencing for large-scale genotyping and *de novo* SNP discovery, was reported [28]. SLAF-seq technology has been used to construct high-density genetic maps for several plant species [29–31]. In this study, we constructed a high-density genetic linkage map containing a number of walnut SNP markers using the SLAF-seq approach, which was based on an F1 population with 84 individuals and two parental individuals. Subsequently, a QTL associated with anthracnose resistance was located and analyzed. The results presented here will aid molecular marker-assisted breeding and walnut resistance genes identification.

## Results

### Analysis of SLAF-seq data and SLAF markers

**Fig. 1:**
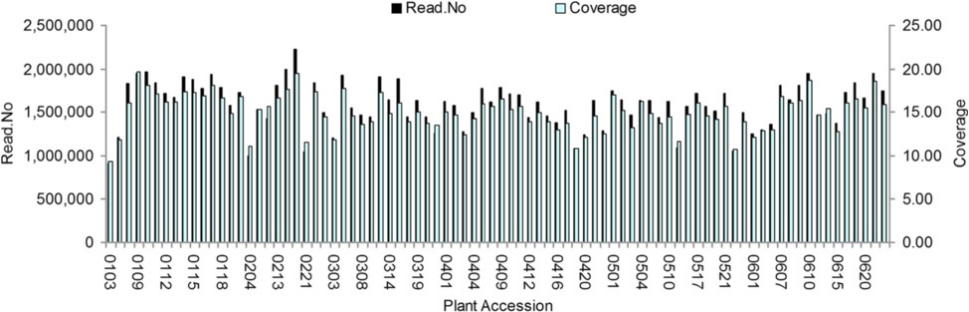
Valid read numbers and coverage for each of the F1 individuals. The x-axes indicate the plant accession including each of the F1 individuals; the y-axis indicates read number and the secondary y-axis indicates cluster coverage

**Fig. 2:**
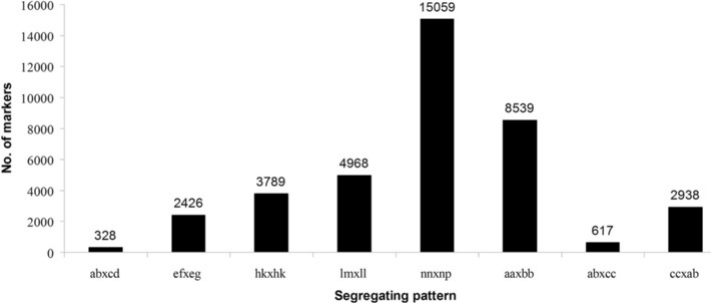
Number of polymorphic SLAF markers for eight segregation patterns. The x-axis indicates eight segregation patterns of polymorphic SLAF markers; the y-axis indicates the number of markers

161.64 M pair-end reads were generated. The Q20 (indicating a 1 % chance of error) was 83 % and guanine-cytosine (GC) content was 41.50 %. The read numbers for SLAFs in the female and male parents were 17,022,710 and 11,890,183, respectively. The numbers of SLAFs in the female and male parents were 133,832 and 119,639, respectively. The average coverage for each marker was 127.19-fold and 99.38-fold in the female and male parents, respectively. In the F1 population, the read numbers of SLAFs in each individual ranged from 918,686 to 2,232,493, with an average of 1,587,259. The numbers of SLAFs ranged from 89,674 to 117,170, with an average of 105,517 in each individual. The coverage ranged from 10.24-fold to 19.05-fold, with an average of 15.04-fold (Fig. 1). The average sequence depths of these SLAFs were 68.06 in the female parent, 56.21 in the male parent and 7.91 in the progeny. 153,820 SLAF markers were obtained, among which 49,174 (31.97 %) were polymorphic (Table 1). All polymorphic SLAFs were then genotyped separately for both parents and all individuals. After discarding SLAF markers that lacked parent information and contained many repeat sequences, 38,664 markers were genotyped successfully and were classified into eight segregation types (Fig. 2). After filtering low-quality SLAF markers, segregation distortion markers and other markers that were not suitable for map construction, 13,635 polymorphic markers were obtained and were sorted to five segregation types (ef × eg: 962, hk × hk: 1,046, lm × ll: 3,501, nn × np: 8,123 and ab × cd: 3).

**Table 1:** SLAF markers mining results

Type	Number of SLAF markers	Ratio	Total sequence depth of SLAF markers	Average sequence depth of SLAF markers
Polymorphic SLAF	49,174	31.97 %	33,804,866	687.45
Non polymorphic SLAF	104,646	68.03 %	65,228,858	623.33
Total	153,820	100.00 %	99,033,724	643.83

Notes: Polymorphic SLAF marker indicates that the marker has two to four alleles; non-polymorphic SLAF marker indicates that the marker has only one allele

### The basic characteristics of the genetic maps

After linkage analysis, 2,577 (Additional file 1, Additional file 2) of the 13,635 markers were used to construct genetic linkage maps, while the other 11,058 markers failed to be mapped onto the genetic map. 761 markers on the map showed significant (P < 0.05) segregation distortion. The average integrity of mapping markers was 100 %. The average depth of the markers was 122.57 in the female parent, 83.62 in the male parent, and 11.23 in the offspring.

**Fig. 3:**
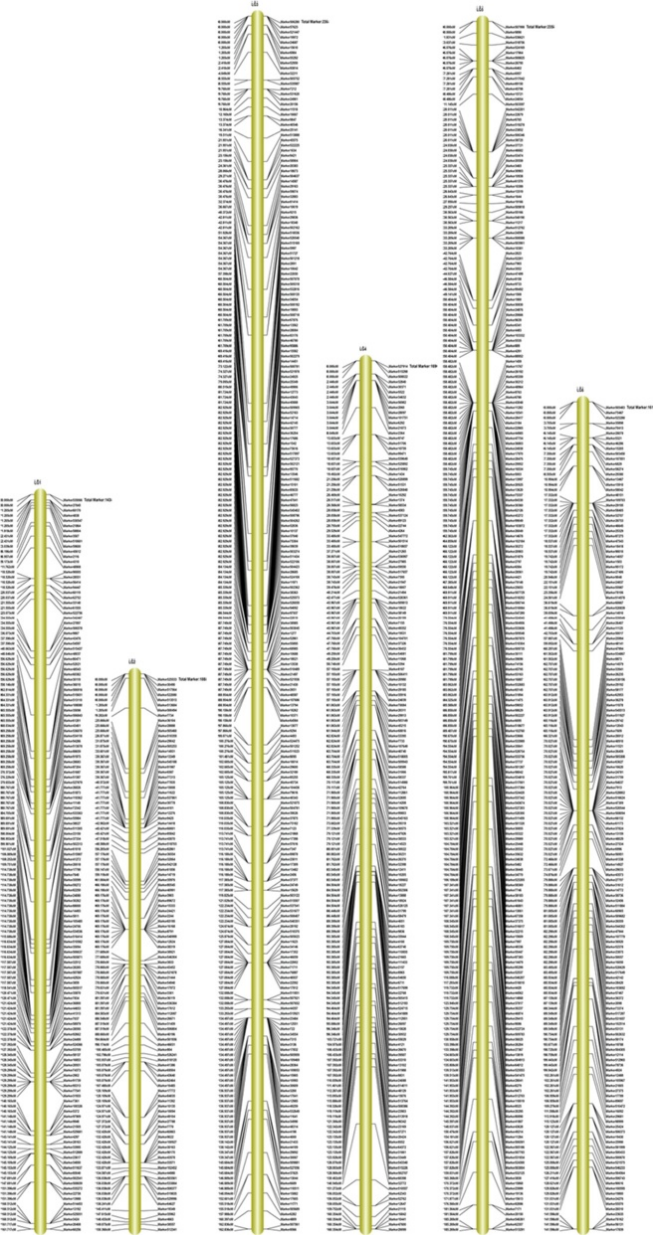
Integrated linkage map for groups 1 to 6 of walnut. The map includes linkage groups 1 to 6 of walnut; the SLAF markers and their locations are shown on the right and left side, respectively

**Fig. 4:**
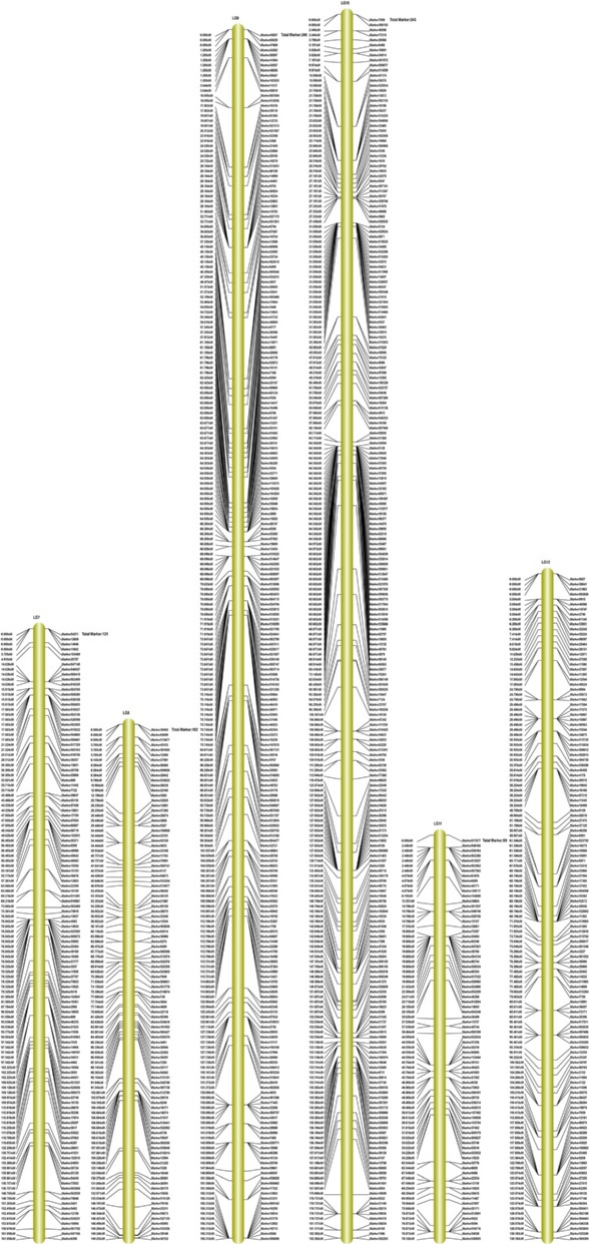
Integrated linkage map for groups 7 to 12 for walnut. The map includes linkage groups 7 to 12 of walnut; the SLAF markers and their locations are shown on the right and left side, respectively

**Fig. 5:**
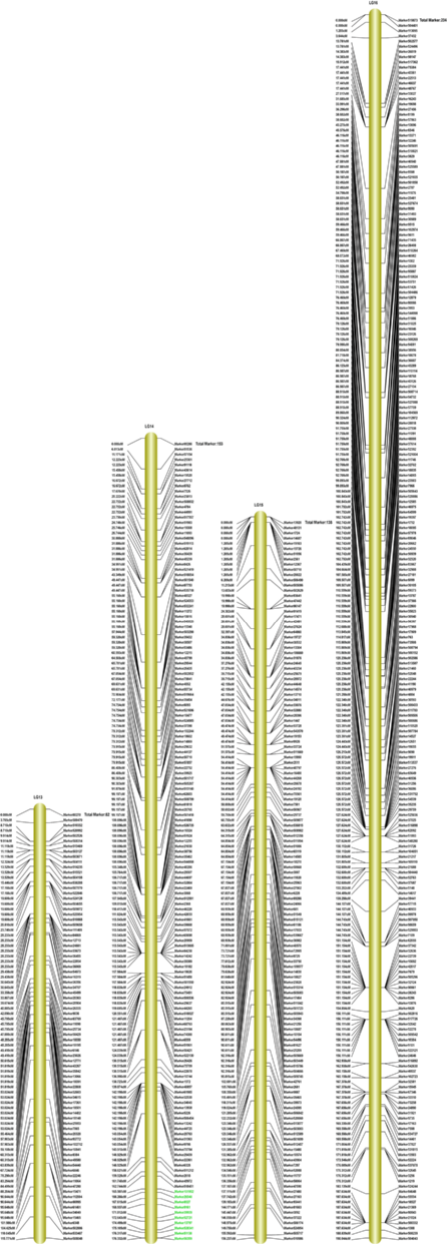
Integrated linkage map for groups 13 to 16 of walnut. The map includes linkage groups 13 to 16 of walnut; the SLAF markers and their locations are shown on the right and left side, respectively. Markers associated with resistance to anthracnose are labeled with green on LG14

Among the 2,577 markers, 2,395 of these fell into 16 linkage groups (LGs) for the female map, 448 markers for the male map, and 2,577 markers for the integrated map (Figs. 3, 4 and 5, Additional file 3). Taking into account the size of all LGs, marker coverage amounted to 2,664.36 cM for the female map, 1,305.58 cM for the male map, and 2,457.82 cM for the integrated map. The average intervals between two adjacent mapped markers were 1.11 cM, 2.91 cM and 0.95 cM for female maps, male maps, and integrated maps, respectively.

All LGs are shown in Table 2: LG10 contained the most markers for the female map (242 markers), LG11 for the male map (80 markers) and LG10 for the integrated map (243 markers). LG11 contained the fewest markers for the female map (one marker), LG3 and LG13 for the male map (one marker) and LG11 for the integrated map (80 markers). The longest LGs were LG10 for the female map (274.38 cM), LG16 for the male map (174.78 cM) and LG16 for the integrated map (190.04 cM). The shortest were LG11 for the female map and the integrated map, and LG3 and LG13 for the male map, with 0.00 cM, 78.12 cM and 0.00 cM, respectively.

**Table 2:** Basic characteristics of the 16 walnut linkage groups

	Number of markers			Size (cM)			Average distance between markers (cM)		
	Female map	Male map	Integrated map	Female map	Male map	Integrated map	Female map	Male map	Integrated map
LG1	142	17	143	163.08	145.13	161.72	1.15	8.54	1.13
LG2	108	9	108	162.19	91.02	150.36	1.50	10.11	1.39
LG3	236	1	236	162.84	0.00	162.84	0.69	0.00	0.69
LG4	169	27	169	175.49	126.56	160.35	1.04	4.69	0.95
LG5	233	5	235	180.84	91.76	185.27	0.78	18.35	0.79
LG6	161	5	161	138.75	23.09	141.60	0.86	4.62	0.88
LG7	121	7	121	194.72	89.02	161.06	1.61	12.72	1.33
LG8	102	69	102	152.96	111.10	149.25	1.50	1.61	1.46
LG9	218	40	240	208.91	65.03	156.31	0.96	1.63	0.65
LG10	242	31	243	274.38	69.25	182.38	1.13	2.23	0.75
LG11	1	80	80	0.00	78.12	78.12	0.00	0.98	0.98
LG12	123	17	132	135.60	31.46	130.18	1.10	1.85	0.99
LG13	82	1	82	115.77	0.00	115.77	1.41	0.00	1.41
LG14	153	31	153	209.41	136.44	176.33	1.37	4.40	1.15
LG15	125	34	138	190.18	72.82	156.24	1.52	2.14	1.13
LG16	179	74	234	199.25	174.78	190.04	1.11	2.36	0.81
Total	2395	448	2577	2664.36	1305.58	2457.82	/	/	/

### Distribution of marker types on the genetic map

**Fig. 6:**
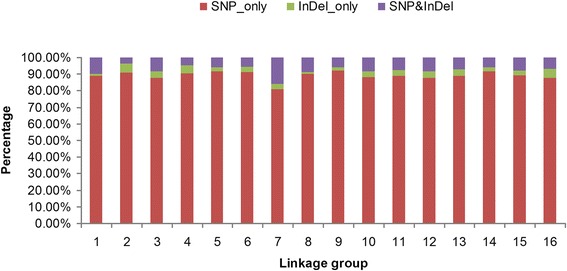
Percentages of diverse types of markers on each linkage group. The x-axis indicates the 16 linkage groups of the integrated map, the y-axis indicates the percentages of three types of markers: ‘SNP_only,’ ‘InDel_only,’ and SNP & InDel on each linkage group

The integrated map had three types of markers: 2300 ‘SNP_only,’ 87 ‘InDel_only,’ and 190 ‘SNP&InDel’. ‘SNP_only’ was the main type, accounting for 89.25 % of the markers on the integrated map. The marker types in each of 16 LGs are shown in Fig. 6. LG2 and LG16 contained the highest percentage of ‘InDel_only’ markers at 5.56 %. LG7 contained the highest percentage of ‘SNP&InDel’ markers and the lowest percentage of ‘SNP_only’ markers, at 15.70 % and 80.99 %, respectively. LG9 contained the highest percentage of ‘SNP_only’ markers at 92.08 %.

5,043 SNP loci were detected among the 2,577 markers on the genetic maps, which correlated with two SNP loci per SLAF marker, and percentages of different SNP types were investigated (Table 3, Additional file 4). Most of the SNPs were transition-type SNPs, containing R (G/A) and Y (T/C) types, accounting for 33.61 % and 34.62 % of all SNPs, respectively. The other SNPs were transversion-type SNPs (31.77 % of total SNPs) containing S (G/C), M (A/C), K (G/T), and W (A/T), with the percentages ranging from 5.23 to 9.66 %.

**Table 3:** Statistics of SNP types on the map

Type	Number	Ratio
S (G/C)	264	5.23 %
M (A/C)	414	8.21 %
K (G/T)	437	8.67 %
W (A/T)	487	9.66 %
R (G/A)	1,695	33.61 %
Y (T/C)	1,746	34.62 %
Total	5,043	100.00 %

Notes: Number indicates the number of each SNP type: R (G/A) and Y (T/C) are transition-type SNPs, and S(G/C), M(A/C), K(G/T) and W(A/T) are transversion-type SNPs

### Visualization and evaluation of the genetic map

The quality of the integrated map was evaluated by haplotype mapping. The population of double exchange and the genotyping errors were reflected in the haplotype map. Haplotype maps were produced for each of the 84 offspring and for the parental controls, using 2,577 SLAF markers, as described by West et al. [32]. The recombination events of each individual were displayed in the haplotype maps visually (Additional file 5). A large proportion of the recombination blocks were distinctly defined. Less than 0.1 % contained no heterozygous fragments, and less than 0.6 % was missing. Except for those that contained a few heterozygosity sites, all LGs distributed uniformity. Therefore, the F1 population was suitable for genetic analysis.

The relationship of recombination between markers from each LG was reflected by a heat map. Pairwise recombination scores for 2,577 markers were used to construct heat maps to evaluate the integrated map quality (Additional file 6). The accuracy of mapping was estimated using the rate of recombination and genetic distance between two markers.

### The QTL analysis of walnut anthracnose resistance

**Fig. 7:**
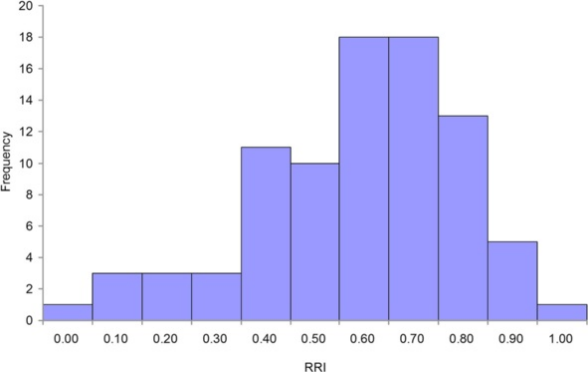
The frequency distribution of the relative resistance index (RRI) of walnut individuals. The x-axis indicates the RRI; the y-axis indicates frequency of the RRI

**Fig. 8:**
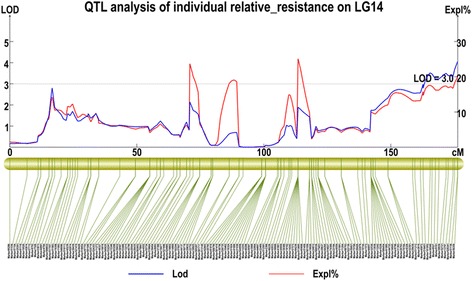
Logarithm of odds (LOD) and percent phenotypic variance explained (PVE) curves on LG14 for anthracnose resistance of walnut. The blue curve indicates LOD scores of SLAF markers against their genetic position on LG14. The red curve indicates the PVE of SLAF markers against their genetic position on LG14. The gray line indicates the threshold LOD score

Based on statistics data about the relative resistance index (RRI) of 86 individuals comprising the paternal parent, maternal parent and F1 offspring (Additional file 7). The RRI of the paternal parent was 0.81, the maternal parent was 0.00. A frequency distribution graph of RRI was constructed and shown in Fig. 7. Further analysis using interval mapping (LOD >3.0) detected one QTL that was associated with resistance to anthracnose, which ranged from 165.51 to 176.33 cM on LG14. Ten markers (marker101952, marker26540, marker8527, marker9161, marker39939, marker52721, marker13797, marker528341, marker51138, marker56359) in this interval that were above the LOD threshold value (LOD > 3) were considered to be linked markers to the anthracnose resistance trait. The phenotypic variance explained by each marker ranged from 16.2 to 19.9 %, with an average of 17.63 %, and their LOD scores varied from 3.22 to 4.04, with an average of 3.54 (Fig. 8).

## Discussion

### Mapping population

The selection of mapping parents is important for constructing high-density map. Establishing a suitable mapping population is the first step in constructing a map and determines its quality. For many agricultural crops, segregating crosses initiated with two contrasting inbred lines, such as the BC, Double haploid (DH), Recombination Inbred Lines (RILs) and F2, have been used commonly for genetic/QTL linkage mapping [33]. For many forest trees, whose biological properties prevent the generation of inbred lines, and therefore, of any advanced crosses. However, because of their high heterozygosis, tree species are able to generate large progeny sets from full-sib or half-sib crosses, and the crosses among their F1s often display substantial segregation [34, 35]. Grattapaglia and Sederoff [36] put forward a so-called pseudo-testcross strategy for linkage mapping in a controlled cross between two parents. In that method, the pseudo-testcross mapping strategy is based on the selection of single dose markers present in one parent and absent in the other. Walnut is a monoecious plant that develops unisexual female and male flowers within separate inflorescences of the same individual. To construct a genetic map and detect QTLs for anthracnose resistance, the F1 population from a cross between walnut cultivar ‘*Yuan Lin*’ (susceptible to anthracnose) and ‘*Qing Lin*’ (resistance to anthracnose) was created by controlled pollination, and resistance to walnut anthracnose caused by *C. gloeosporioides* of the individuals was evaluated by RRI (Fig. 7, Additional file 7). RRIs of the female and male parents were 0.00 and 0.81, respectively; RRIs of offspring ranged from 0.06 to 0.91, with an average of 0.54. The coefficient of variation (CV) was 36.24 %. The results showed that the anthracnose resistance of the F1 population was continual and had quantitative character of inheritance that would be controlled by multiple genes.

### The segregation of SLAF markers

SLAF-seq can generate large numbers of markers and provide a good platform for species without a reference genome sequence, such as walnut [28, 29]. In this study, SLAF-seq was applied to determine the genome sequence of walnut and a total of 161.64 M pair-end reads were generated. Among the 153,820 SLAF markers obtained, 49,174 SLAFs were polymorphic (Table 1). 13,635 polymorphic markers were successful genotyped and sorted into five segregation types (ef × eg: 962, hk × hk: 1,046, lm × ll: 3,501, nn × np: 8,123 and ab × cd: 3). Ratios of segregation types were different in the populations, which indicated that the recombination frequency of different chromosomes was inconsistent during the period of meiosis [22].

After linkage analysis, 2,577 SLAF markers were used to construct genetic maps. ‘SNP_only’ markers were predominant (89.25 %) on the integrated map (Fig. 6). 5,043 SNP loci were detected among the 2,577 markers, corresponding to two SNP loci per SLAF markers (Table 3, Additional file 4). You et al. [2] identified 13,439 genome-wide SNPs in walnut (‘Chandler’ cultivar) using bacterial artificial chromosome end sequences (BESs). Our results indicated that SLAF-seq is an effective method to develop SNP markers for walnut.

Segregation distortion is a widespread and common phenomenon in many plants, which deviates the frequency of alleles from representative Mendelian ratio [37, 38]. The phenomenon may be related to the population type and environmental factor [39, 40]. Seven hundred sixty one markers showed significant (P < 0.05) segregation distortion, accounting for 29.53 % of the markers on the integrated map. Bradshaw and Stettler [41] discovered that segregation distortion markers had almost the same mapping efficiency as segregation normal markers. Discarding segregation distortion markers would possibly cause the loss of massive amounts of information and decrease the coverage of the genome [42]. Zhang et al. [43] and Xu [44] showed that segregation distortion markers did not have an effect on QTL mapping. Therefore, segregation distortion markers could be used to construct the genetic map.

### Linkage mapping

A high-density genetic linkage map aids QTL mapping for important economical traits. Genetic maps of walnut were reported by Fjellstrom et al. [1] and Woeste et al. [18]. However, because of the lack of a large number of molecular markers with sufficient sequence information, many QTLs for target traits cannot be accurately detected. Here, among the 2,577 markers, 2,395 of these fell into 16 LGs for the female map, 448 markers for the male map, and 2,577 markers for the integrated map, the average intervals between two adjacent mapped markers were 1.11 cM, 2.91 cM and 0.95 cM for the female map, the male map, and the integrated map, respectively (Table 2). Thus, the density of the map was higher than that of previous maps. The 16 LGs corresponded to the haploid genome of walnut, which has 16 chromosomes [1, 2, 45]. The quality of genetic map was evaluated by haplotype map (Additional file 5), and the relationship of recombination between markers from each linkage group was reflected by heat map (Additional file 6). To avoid sequencing or mapping errors, High Map Strategy [46] was used to order and correct SLAF markers and for genotyping. The results indicated the map is efficient, accurate and saturated. Li et al. [47] and Doerge [48] considered that 10 cM marker density is sufficient to provide an accurate estimation of QTL positions for a population size between 100 ~ 200. Consequently, we constructed the linkage map that met these requirements for QTL analysis.

### QTL mapping for anthracnose resistance

QTL mapping for anthracnose resistance in other plants have been studied. Lee et al. [49] obtained seven QTLs for resistance to *C. acutatum* ‘KSCa-1’ and *C. capsici* ‘ThSCc-1’ in pepper (*Capsicum* spp.), two of which were major QTLs. Petro et al. [50] detected nine QTLs associated with anthracnose resistance, which explained 7.0–32.9 % of the phenotypic variance in water yam (*Dioscorea alata* L.). Woeste et al. [51] obtained a RAPD marker linked to hypersensitivity to the cherry leafroll virus from a walnut BC population using bulked segregant analysis (BSA). In the present study, according to the high-density genetic map for walnut, one QTL for walnut anthracnose resistance was identified. The interval of this QTL ranged from 165.51 cM to 176.33 cM on LG14, and contained ten markers associated with anthracnose resistance (above LOD > 3), that the average intervals between two adjacent markers was 1.08 cM. The phenotypic variance explained by each marker ranged from 16.2 to 19.9 % (Fig. 8). A high LOD threshold can decrease false positives effectively, and a LOD threshold of 2–3 can control the value of α to within 0.05 [52, 53]. The 10 markers that were detected belonged to the segregation type hk × hk, which might indicate that the resistance to anthracnose was inherited from the parents. In future experiments, we will use the QTL to develop efficient markers associated with walnut anthracnose resistance and to accelerate walnut breeding project.

## Conclusions

High-density genetic linkage maps of walnut were constructed using the SLAF-seq method. The maps contained 2,577 polymorphic markers, from which 5,043 SNP loci were detected. One QTL was identified for resistance to anthracnose on LG14. Our results provide an important theoretical basis for walnut molecular assisted breeding and resistance gene identification.

## Methods

### Plant material and DNA extraction

The cross combination of walnut cultivar ‘*Yuan Lin*’ (susceptible to anthracnose, maternal) and cultivar ‘*Qing Lin*’ (resistance to anthracnose, paternal) was designed and hybridized with bags in the spring of 2002, and harvested in the autumn. Eighty-four F1 individuals comprising the mapping population were planted in a 2 m × 3 m field, which was seeded in the spring of 2003. All plant materials were grown at the Forestry Experimental Station of Shandong Agricultural University, Taian, Shandong, China (N36°10′19.2′′, E117°09′1.3′′). Young leaves from the two parents and the F1 progeny were collected, frozen in liquid nitrogen and genomic DNA was extracted using the cetyltrimethylammonium bromide (CTAB) method described by Fjellstrom et al. [1]. A NanoDrop™ 2000/2000c (Thermo Scientific, Waltham, MA, USA) was used to detected DNA concentrations.

### The evaluation of walnut anthracnose resistance

Evaluation of anthracnose resistance was carried out for the parental parent, maternal parent and F1 progeny using a conidial suspension (1 × 10^6^ conidia mL^−1^) of *C. gloeosporioides* isolate WSG1 (GenBank accession of its internal transcribed spacer: HQ828069) that was needle inoculated onto ~100 healthy leaves in early June, 2012. About 10 days after inoculation, the severity of disease symptoms was recorded according to a plant disease index (DI) based on the percentage of necrotic area. Grading standards of disease severity were described by Fang et al. [54]. The DI was calculated by the following equation:

$$ \mathrm{D}\mathrm{I} = \sum\ \left(\mathrm{Number}\ \mathrm{of}\ \mathrm{each}\ \mathrm{grade} \times \mathrm{Grades}\right)\ /\ \left(\mathrm{Total}\ \mathrm{number} \times \mathrm{highest}\ \mathrm{grade}\right) \times 100. $$

The RRI was used to evaluate disease resistance of the F1 population and parents on the basis of the disease index [11], and calculated according to the following formula:

$$ \mathrm{R}\mathrm{R}\mathrm{I}=1 - \mathrm{relative}\ \mathrm{disease}\ \mathrm{index}\ \left(\mathrm{R}\mathrm{D}\mathrm{I}\right); $$

$$ \mathrm{R}\mathrm{D}\mathrm{I} = \mathrm{disease}\ \mathrm{index}\ \mathrm{per}\ \mathrm{plant}/\mathrm{highest}\ \mathrm{disease}\ \mathrm{index} $$

### SLAF library construction and high-throughput sequencing

In this experiment, two SLAF libraries were constructed using the pre-designed scheme with two enzymes to generate more SLAF markers. The procedure was performed as follows. Genomic DNA from each sample (84 individuals and two parents) was digested at 37 °C with *Mse* I, which was called SLAF library 1 and *Hae* III as SLAF library 2, [New England Biolabs (NEB), Ipswich, MA, USA]. Incubation with the Klenow Fragment (3′ → 5′ exo–) (NEB) and dATP (NEB) at 37 °C provided a single-nucleotide A overhang for the digested fragments, and then T4 DNA ligase (NEB) was used to ligate the A-tailed fragments and the Duplex Tag-labeled Sequencing adapters (PAGE purified, Life Technologies, USA). PCR reactions were performed with dNTP mix (NEB), diluted restriction-ligation DNA samples, Q5® High-Fidelity DNA Polymerase (NEB), blunting buffer (NEB) and primers, whose sequences were 5′-AATGATACG GCGACCACCGA-3′ and 5′-CAAGCAGAAGACGGCATACG-3′ (PAGE purified, Life Technologies). Reaction products were purified using Agencourt AMPure XP beads (Beckman Coulter, High Wycombe, UK), and pooled into one pool. Pooled samples were separated on a 2 % agarose gel. Fragments (SLAF1) of 389–443 bp and fragments (SLAF2) of 309–457 bp (with indexes and adaptors) were purified using a QIAquick Gel Extraction Kit (Qiagen, Hilden, Germany). The purified products were sequenced on the Illumina HiSeq 2500 system (Illumina, Inc; San Diego, CA, USA) in accordance with the manufacturer’s recommendations [28].

### SLAF-seq data analysis and genotyping

The SLAF-seq data grouping and genotyping were performed as described in detail by Sun et al. [28]. Raw reads were assigned to 86 individuals according to the barcode sequences. To reduce computational intensity, identical reads were merged together, and sequence similarity was detected using one-to-one alignment by BLAT [55] (−tileSize = 10 -stepSize = 5). High quality reads with quality scores >20 were identified for quality control. Sequences with over 90 % identity among all SLAF paired-end reads that clustered together were grouped into one SLAF locus [28]. Alleles were defined in each SLAF using the minor allele frequency (MAF) evaluation. As a diploid organism, walnut could have no more than four alleles for one SLAF marker; therefore, SLAF markers with more than four alleles or one allele were filtered out. SNPs or Indels were defined by difference from high depth fragment. SLAFs with two, three or four tags were regarded as polymorphic SLAFs, and were sorted into eight segregation types (ab × cd, ef × eg, ab × cc, cc × ab, hk × hk, lm × ll, nn × np, aa × bb). The segregation type ‘ab × cd’ represent that the two alleles of one marker are different in both parents. Most of polymorphism loci between the parents in each of the SLAF were SNP types, which means a polymorphism SLAF may contain one or more SNPs. Each SLAF marker was filtered and subjected to quality assessment many times; low quality markers containing less than three SNPs and average depth of each sample below 3 were discarded.

### Segregation distortion analysis and Linkage map construction

The chi-square test was used to calculate marker segregation ratios. Markers showing significant (P < 0.05) segregation distortion were initially eliminated from the map construction and were then added later as accessory markers. A region of segregation distortion was defined as a region with more than three adjacent loci on the map that showed significant (P < 0.05) segregation distortion [56]. Many genotyping errors and deletions may be caused by NGS and can have an effect on the quality of high-density linkage maps; therefore, SLAF markers and genotyping errors within LGs used High Map Strategy to order and correct them [46]. SLAFs markers were ordered using a detailed maximum likelihood (ML) algorithm [57] and genotyping errors were corrected with the SMOOTH algorithm [58]. All linkage groups were created as follows: we obtained an initial marker order according to the relationship between ordered markers, and the SMOOTH algorithm was used to correct genotyping errors or deletions, after that ML was used to order the map. The newly ordered genotypes were then corrected by the SMOOTH again. After four or more cycles, high-density linkage maps were obtained. The Kosambi mapping function was used to calculate the map distances.

### QTL analysis

According to the constructed 16 LGs of the integrated map, the relative resistance index was analyzed by Map QTL5.0 software [59], and interval mapping was used to detect QTL loci. After 1000 permutation tests, we chose a LOD > 3.0 (p ≥ 0.95) as the threshold to detect QTLs associated with resistance to anthracnose in walnut [60].

## Availability of supporting data

The sequence data for 86 samples produced in this study have been deposited in the NCBI SRA database (accession number: SRR2131213). The additional files data supporting the results of this article are available in the LabArchives repository (https://mynotebook.labarchives.com/share/Additional%2520files/MC4wfDk4ODAwLzAvVHJlZU5vZGUvNDE2NjE5Mjk0M3wwLjA=).

## Additional files

Additional file 1:
**Details of markers sequences.** Sequences of each SLAF marker on the maps. (XLS 992 kb) Additional file 2:
**Details of markers genotypes for F1 individuals.** Genotypes of each SLAF marker on the maps for F1 individuals. (XLS 3163 kb) Additional file 3:
**SLAF markers on the 16 linkage groups (LGs).** The SLAF markers and their locations in each LG on the maps. (XLS 404 kb) Additional file 4:
**Sequences of discovered SNPs.** SNP loci of every marker on the maps are marked in the lowercase. (XLS 992 kb) Additional file 5:
**Haplotype map of the integrated maps.** Each row represents a marker. Markers are ranked in accordance with the map order. Each two columns represent an individual; blank columns are used between two individuals. The first and second columns represent the paternal and maternal chromosome, respectively. Green and blue represent the first and second allele from the parents, respectively. White represents the source of alleles that cannot be judged. Gray represents deleted alleles. (XLS 1321 kb) Additional file 6:
**Heat map of the integrated maps.** Markers of each row and column are ranked according to the map order; each small square represents the rate of recombination (r) between two markers. The domain of r is [0.0, 0.5]. The color varies from yellow to red if r_Min_ ≤ r ≤ 3/8r_Max_. The color is red if r = 3/8r_Max_. The color varies from red to purple if 3/8r_Max_ < r < 3/4r_Max_. The color is purple if 3/4r_Max_ ≤ r ≤ r_Max_. Gray represents deletion. (XLS 620 kb) Additional file 7:
**Relative resistance index of walnut individuals.** Statistics of data concerning the relative resistance index (RRI) of 86 individuals containing paternal parent, maternal parent and F1 offspring. (XLS 23 kb)

## Abbreviations

SLAF-seq: Specific length amplified fragment sequencing
QTL: Quantitative trait locus
SNP: Single nucleotide polymorphism
LG: Linkage group
RGAs: Resistance gene analogs
R gene: Resistance gene
RFLP: Restriction fragment length polymorphism
RAPD: Random amplified polymorphic DNA
SSR: Simple sequence repeat
ISSR: Inter-simple sequence repeat
BC: Backcross
BAC: Bacterial artificial chromosome
NGS: Next generation sequencing
RRLs: Reduced-representation libraries
CRoPS: Complexity reduction of polymorphic sequences
GBS: Genotyping by sequencing
RAD-seq: Restriction-site associated DNA sequencing
GC: Guanine-cytosine
RRI: Relative resistance index
LOD: Logarithm of odds
DH: Double haploid
RIL: Recombination Inbred Line
BESs: Bacterial artificial chromosome end sequences
MAS: Marker-assisted selection
CTAB: Cetyltrimethylammonium bromide
DI: Disease index
RDI: Relative disease index
NEB: New England Biolabs
MAF: Minor allele frequency
PVE: Phenotypic variation explained
CV: Coefficient of variation
BSA: Bulked segregant analysis
